# Light and temperature effects on bioactivity in diatoms

**DOI:** 10.1007/s10811-015-0631-4

**Published:** 2015-06-05

**Authors:** Richard A. Ingebrigtsen, Espen Hansen, Jeanette Hammer Andersen, Hans Christian Eilertsen

**Affiliations:** Department of Arctic and Marine Biology, Faculty of Biosciences, Fisheries and Economics, UiT - The Arctic University of Norway, Tromsø, N-9037 Norway; Marbio, UiT - The Arctic University of Norway, Tromsø, N-9037 Norway

**Keywords:** Diatoms, Bioactivity, OSMAC method

## Abstract

Isolates of five pelagic North Atlantic marine diatoms (Bacillariophyceae): *Attheya longicornis*, *Chaetoceros socialis*, *Chaetoceros furcellatus*, *Skeletonema marinoi* and *Porosira glacialis* were cultivated in large photobioreactors at two light and two temperature regimes to test if this affected bioactivity. We screened for bioactivity in assays representing five different therapeutic areas: diabetes II (PTP1b), cancer (melanoma cells, A2058), anti-oxidants (FRAP), immunomodulation (TNFa) and anti-infection (MRSA, *Enterococcus faecalis*, *Staphylococcus aureus*, *Escherichia coli* and *Pseudomonas aeruginosa*). All the diatom strains showed activity in two or more assays. We detected differences in bioactivity both between species and within species cultivated with different light and temperature regimes. Our results demonstrate the potential for a more exhaustive exploitation of diatom metabolites that can be obtained by manipulation of the cultivation conditions.

## Introduction

Marine diatoms usually represents less than 0.1 % of the Earth’s autotrophic biomass but are, due to high growth rates, responsible for more than 40 % of the total primary production (Nelson et al. 1995; Field et al. 1998). Marine diatoms have evolved in a habitat abundant in specialized phytoplankton grazers, pathogenic bacteria and viruses. In order to survive, the diatoms have developed mechanisms to avoid, tolerate or deter them (Smetacek 2001). There are numerous ways to increase fitness ranging from mechanical protection in the form of a tough glass frustule with pointy spines (van den Hoek et al. 1995; Hamm et al. 2003) to production of allelopathic grazing deterrence chemicals (Buttino et al. 2008; Ianora et al. 2003; Pohnert 2002). Diatoms have a high chemical diversity, and the chemical composition is affected by the surrounding physical environment, i.e. light, temperature and inorganic nutrient availability (Harrison et al. 1977; Huseby et al. 2013). In addition to this, strains of the same species may respond chemically different to the environment, as well as the developmental stage of the culture may affect the chemical composition of the cells (Lakeman et al. 2009; Barofsky et al. 2010).

While marine bacteria, porifera, molluscs and seaweeds have proven to be important sources of new molecules and potential drugs, diatoms are underrepresented in terms of reported bioactive marine compounds (Blunt et al. 2009, 2010, 2011; Folmer et al. 2010; Bull and Stach 2007). In fact, only a handful of diatoms seem to have been investigated, and only a few diatom-derived bioactive substances have been reported (Rowland et al. 2001; Bergé et al. 1999; Carbonnelle et al. 1999; Prestegard et al. 2009; Zupo et al. 2014; Moreau et al. 2006; Haimeur et al. 2012). And northern or arctic diatoms do not appear to have been investigated for biodiscovery purposes.

Cultivation of marine microorganisms, especially marine bacteria, lately has attracted much attention. Variations in cultivation conditions may turn metabolic pathways on and off and thus trigger synthesis of chemicals. In a study by Zupo et al. (2011) on the diatom *Cocconeis scutellum*, it was shown that optimal growth and production of a specific bioactive compound varied with cultivation conditions. A similar approach called OSMAC (One Strain Many Compounds) that involve systematically testing various fermentation conditions has proven successful when searching for bioactive compounds from bacteria (Bode et al. 2002). This strategy also allows for reproduction of experiments if they yield interesting material. Furthermore, large-scale production of biomass in bioreactors can be realized (Tredici 2004). However, few diatoms have so far been utilized for biodiscovery purposes, and this has mainly been attributed to difficulties in large-scale cultivation (Lebeau and Robert 2003). Furthermore, the recent advances in genome sequencing have highlighted that diatom genomes are very variable between genera and contain very diverse genes (Armbrust et al. 2004). In the present study, we attempted to mass cultivate northern diatoms and to alter production of diatom secondary metabolites by manipulating the two key variable irradiance and temperature.

## Materials and methods

Non-axenic clonal cultures of five different planktonic marine diatoms were established from single cells or single chains from samples collected in the Barents Sea and along the coast of northern Norway. Isolation method applied was a combination of microscope aided manual capillary pipetting and serial transfer (i.e. transferring a single cell or chain to a well in a 24-well chamber (Nunc) filled with autoclaved seawater and serial transferring it 5–10 times to rinse it). Although non-axenic, bacterial numbers were very low. The culture collection and innocula for mass cultures were cultivated in pasteurized seawater from 10-m depth in Tromsøysund with 4 mL L^−1^ of Guillard’s f/2 × 50 marine water enrichment solution (Sigma–Aldrich). The species *Attheya longicornis*, *Chaetoceros furcellatus*, *Chaetoceros socialis*, *Porosira glacialis* and *Skeletonema marinoi* were identified using a combination of morphological and molecular methods (18 and 28 s rDNA) as described by Degerlund et al. (2012).

All cultivations took place in temperature (±1 °C) and irradiance controlled rooms (Table 1). The diatoms were cultivated as semi-continuous cultures (i.e. by harvesting daily a fraction of the culture and subsequently replacing it with fresh nutrient replete medium) in 630-L transparent plexiglas columns. This cultivation mode allowed us to keep the diatom population in exponential growth phase throughout the cultivation. Large cultivation volumes were necessary to obtain the amount of biomass needed to perform the assays included in the screening and to still have biomass left for re-testing, isolation and structure elucidation of potential bioactive compounds. The cultivation took place in Millipore filtered seawater (0.22-μm pore size) with nutrients (0.25 mL L^−1^ Substral™ (Scotts Company (Nordics) A/S)) and silicate (Na_2_O_3_Si·9H_2_O, Sigma–Aldrich) added to a final concentration of 12.3 μmol L^−1^. The reason for choosing Substral, which is a product mainly used as a fertilizer for household plants, was that it is cheap and that we found it to be an adequate nutrient source (see Table 2 for overview of main components in Substral and Guillard’s f/2). Osram L 58W/954 Daylight tubes placed around the columns provided illumination, and the photoperiod was 14:10 (light/dark). Photosynthetical active radiation (PAR) was measured with a QSL-100 scalar irradiance meter (Biosperical Instruments Inc.). Appropriate temperature and light ranges for the five strains were tested in pilot experiments. Note that the mean light intensity the cells received decreased during the experiments due to increased self-shading, as the cultures became denser.

**Table 1:** Light and temperature regimes applied for the cultivations

Species	Weighted mean temperature (°C)		Weighted mean scalar irradiance (μmol photons m^−2^ s^−1^)	
	High	Low	High	Low
*A. longicornis*		4.3		76
	9			30
	9		130	
*C. furcellatus*		4.3		30
		5	130	
	8.5			68
	9		130	
*C. socialis*		4.5	130	
	6.6			35
*P. glacialis*		3.3		93
		4.5	160	
	6.5			80
	7.1		135	
*S. marinoi*		5.6	115	
	8.5			55
	8.7		93	

Each strain was cultivated at a high and a low light and temperature regime. The light intensities and temperatures applied for cultivation were different between the strains due to differences in their temperature and irradiance tolerances. Light intensities given are start values. Note that “high” temperature in this context with e.g. the obligate cold-water species *P. glacialis* is only around 7 °C

**Table 2:** Main components of Substral™ (Scotts Company (Nordics) A/S)) and Guillard’s f/2 marine nutrient enrichment solution (Sigma–Aldrich)

Substral		Guillard’s f/2	
Component	Contents (mg mL^−1^)	Component	Contents (mg mL^−1^)
Nitrate	39.24	Sodium nitrate	75
Ammonia	32.08	Sodium phosphate	4.41
Phosphate	15.44	EDTA disodium·2H_2_O	4.36
Trace minerals/	Trace amounts	Ferric chloride·6H_2_O	3.15
Vitamins	Trace amounts	Trace minerals/	Trace amounts
		Vitamins	Trace amounts

All cultures were aerated at a rate of ca. 2.5 L min^−1^. Biomass samples were prepared by careful harvesting (using gravity only) onto 10–20 μm plankton nets. This procedure usually lasted between 3 and 4 h. To further concentrate the biomass, it was scraped onto a sieve with 10–20-μm mesh. The diatom concentrates were then centrifuged at 2500 rpm for 10 min, and the pellet and supernatant were separated into 50-mL Falcon tubes and flash frozen in liquid N_2_ before being transferred to a biofreezer (−80 °C). Only the pellet samples were investigated in the present investigation. The cultures were inspected for healthiness and contamination using an inverted Leica light microscope at ×400 magnification before and during cultivation and prior to harvesting. The minimum volume wet pellet needed in further analysis was ca. 25 mL.

## Extraction

Freeze-dried biomass samples were ground and extracted twice with 80 % aqueous methanol (Merck, diluted with Milli-Q H_2_O), using approximately 20 and 10 mL 80 % aqueous methanol per gram dry material in the first and second extractions, respectively. The use of 80 % aqueous methanol has been shown to give high extract yields (Sultana et al. 2009), and it also aided in reducing salt content in the sample. The combined extract was filtered through a Whatman no. 3 filter (Ø 125 mm), and the filtrate was evaporated under reduced pressure using a rotavapor (Laborota 4002, Heidolph). The extract was stored at −23 °C until fractionation.

## Fractionation

Aliquots of the algal extracts (ca. 200 mg) were dissolved in 1 mL 50 % aqueous acetonitrile (with Milli-Q H_2_O) to get a phase separation with a polar (salt in sample contributed to this) and a slightly less polar phase. Only the less polar phase was investigated in this study. The two phases were split into two Eppendorf tubes, centrifuged for 30 min at 13,000 rpm and transferred into glass tubes for HPLC. The two sample phases were fractionated with reversed phase (RP) semi-preparative HPLC into 40 fractions (1 min fractions, see Table 3). Solvents were A: Milli-Q H_2_O and B: Acetonitrile, both with 0.1 % formic acid (Sigma–Aldrich). Flow was 6 mL min^−1^. The semi-prep system consisted of a Waters 600 pump, 2767 Sample Manager, 2996 photodiode array detector and 3100 mass detectors. The column used was a Waters XTerra C18 10 μm, 10 × 250 mm. The fractions were transferred to deepwell plates, dried in a SpeedVac and stored at −20 °C until screening. Diatom extracts with fractions that were active in an assay will hereafter be referred to as “hits”.

**Table 3:** Solvent gradient used for HPLC separation: 40 fractions and 1 min. fractions

Time (min)	Solvent A (%)	Solvent B (%)	Flow rate (mL min^−1^)
0	80	20	6
2	80	20	6
30	0	100	6
40	0	100	6

Solvent A is Milli-Q H_2_O, and solvent B is acetonitrile

## Antioxidant assay (FRAP)

The ferric-reducing ability of plasma (FRAP) assay was carried out in clear 96-well plates according to methods described in Benzie and Strain (1996) and measured at 595 nm in a DTX 880 Multimode Detector (Beckman Coulter, USA). A calibration curve was produced in each plate by adding known concentrations of Trolox (Sigma–Aldrich) (expressed as μM Trolox equivalents). Activity threshold was set to be above 100 μM TE (Trolox equivalents).

## Antibacterial assays

Bacterial strains were seeded from agar into 8 mL of the appropriate enrichment media. All strains used were from the American Type Culture Collection (ATCC), ATCC number in parentheses: *Staphylococcus aureus* (25923), Methicillin-resistant *S. aureus* (33591), *Escherichia coli* (25922), *Pseudomonas aeruginosa* (27853) and *Enterococcus faecalis* (29212). Media used was Mueller–Hinton broth, except for *E. faecalis*, which was cultivated in Brain Heart Infusion. After 1 day, 2 mL was transferred into 25 mL medium and incubated until in log-phase and then diluted 1:1000 in enrichment medium before adding 50 μL suspension to each well (96-well plates). Organic fractions for bacterial testing were dissolved in 7.5 μL DMSO (Sigma–Aldrich) and mixed with 1500 μL ddH_2_0. Fifty microliters from each fraction was added in duplicate into five individual clear 96-well plates (one for each bacterial strain). Both positive and negative controls were used, while gentamicin was applied as a control of the setup and the precision between experiments. The plates were incubated for 24 h at 37 °C. If visible coloration was seen prior to addition of bacterial suspension, then absorbance (OD600 nm) was measured to allow a post-compensation. At the end of the experiment, the plates were read in a 1420 Multilabel Counter VICTOR^3^. Activity threshold was set to be OD_600_ readings below 0.05.

## Diabetes assay (PTP1B)

The enzymatic PTP1B assay was performed at 37 °C using fluorescent 6,8-difluoro-4-methylumbelliferyl phosphate (DiFMUP: VWR, Leuven, Belgium) as substrate and recombinant human PTP1B (Calbiochem) enzyme. Organic fractions were dissolved in 22.5 μL DMSO, and then 477.5 μL assay buffer was added. Aliquots containing 25 μL of each fraction solution mixed with 50 μL PTP1B (1.56 ng well^−1^) were added in triplicate into black 96-well plates and incubated 30 min in the dark. After incubation, 25 μL 10 μM DiFMUP was added and the plates incubated a further 10 min in the dark before fluorescence was measured with a DTX 880 Multimode Detector (Beckman Coulter, USA) at excitation (λ) 360 nm and emission λ 465 nm wavelength. Assay buffer was used as negative control while the positive control consisted of a 160 μM solution (in assay buffer) of PTP inhibitor IV (Calbiochem). Percentage inhibition was calculated by comparing measured values to the controls, and the activity threshold was set to be below 30 % activity.

## Cellular assays

### Immunomodulating assays

THP-1 cells were used in the immunomodulation assays. These macrophages, when activated, produce various cytokines such as tumour necrosis factor α (TNFα) that promote inflammation (Guedes Catarina et al. 2013). In the immunosuppressive assay, extracts that induced inhibition of lipopolysaccharide (LPS, Sigma–Aldrich) induced TNFα expression were regarded active when inhibition was above 50 % compared to the LPS control (without extract). The immunostimulatory variant of the assay was identical, except for not adding LPS to the samples. If expression of TNFα increased in this assay, it was likely caused by the extract. The activity threshold in the immunostimulatory assay was set to above 10 % increase in TNFα expression compared to the LPS control. The assays were performed as described in Lind et al. (2013) using sandwich ELISA to measure cytokine production in the supernatant. Immunology assays were only performed with extracts from *P. glacialis*, *C. furcellatus* and *A. longicornis*.

### Cell viability assay (MTS assay)

HPLC fractions were dissolved in RPMI1640 (BioChrome) and tested against the adherent human melanoma cancer cell line A2058 (ATCC: CRL-11147TM). Cells were seeded in 96-well microtiter plates at 2000 cells/well in Roswell Park Memorial Institute 1640 (RPMI) medium with 10 % foetal bovine serum and 10 μg mL^−1^ gentamicin. They were incubated at 37 °C in a humidified atmosphere with 5 % CO_2_ and 95 % air. The cell line was incubated overnight before addition of fractions and incubation time was 72 h. Viability was determined with the colorimetric assay MTS. After the 72 h exposure, 10 μL Cell Titer 96 Aqueous One Solution (Promega, USA) was added to each well and the plates subsequently incubated for 1 h before being measured in a DTX 880 Multimode Detector (Beckman Coulter) at 485 nm. Cells incubated in RPMI medium was used as positive control and cells treated with Triton X-100 (Sigma–Aldrich) reagent as a negative control. All experiments were performed in triplicate. Activity threshold was set to be below 50 % survival.

## Statistical analysis

Fisher’s exact tests and chi-square tests were run in Excel for Mac 2011 v. 14.4.9. Figures were made using ggplot2 for R (Wickham 2009).

## Results

**Fig. 1:**
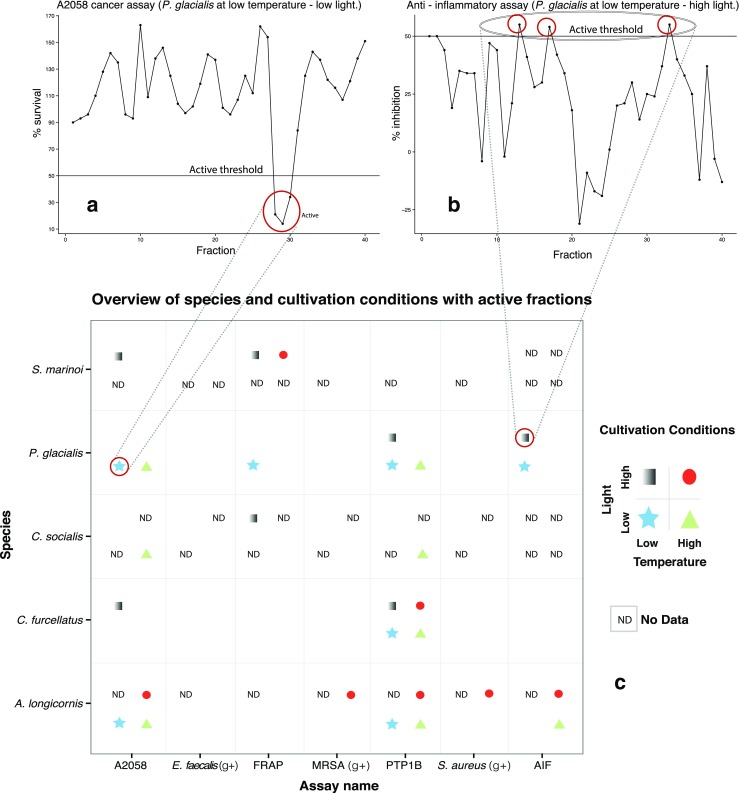
The two *upper* figures show examples of activity profiles from two different assays, anti-cancer assay to the *left* (**a**) and the anti-inflammatory assay to the *right* (**b**), with cutoff *lines* shown (active threshold). If the activity measured was above (as in the anti-inflammatory assay) or below (as in the anti-cancer assay), it is recorded as “active” or a “hit”. Presence/absence of the four geometric *shapes* in the species/assay intersects in the *lower* figure (**c**) depicts hit/no hit at a given cultivation condition, except “ND” indicating no data from the given cultivation condition. Thus, lack of symbol in the given species vs. assay box denotes no hit

We managed to keep healthy cultures in large volumes long enough to produce and harvest enough biomass to do the bioactivity screening and still have some grams of material left. The minimum diatom biomass needed to make enough extract for fractionation and bioactivity testing was circa 25 mL wet pellet. Table 4 presents the bioactivity test results with the number of hits, weakly active and inactive fractions per species in the nine assays. The overall “hit rate” (fractions active in an assay vs. inactive) in this experiment was 1.9 % and if counting in the weak active it was 2.6 % (see Table 5). Of all 3200 anti-bacterial tests, only 6 were active or weakly active against bacteria (0.19 %), while 11.6 and 4.7 % of all tests in the diabetes and anti-cancer (melanoma) assays, respectively, showed activity or weak activity. Figure 1 is a graphical representation of the same data where it is shown which species and cultivation conditions produced hits in the assays applied in addition to examples of two activity profiles. Extracts from all species were active in two or more assays. For the individual assays, we observed one or more hits in most assays except in the *E. coli*, *E. faecalis*, *P. aeruginosa* and the immunostimulatory assays that yielded no hits. All species except *S. marinoi* had extracts with activity in the diabetes assay (PTP1B), while only *A. longicornis* was active against any bacteria tested (MRSA and *S. aureus*). A chi-square test of independence of species, hits and inactive was significant (*P* = 0.0008), indicating independence (*S. marinoi* was not included in this chi-square test since it had only 5 hits). Table 6 is a Fisher’s exact test matrix where the number of active and inactive from each species is tested against all the other species. All species were significantly different from at least one of the other species (*P* ≤ 0.05) in number of hits in relation to number of inactive. The *P* value of each of these tests is shown in Table 6.

**Table 4:** Overview of all assay results from all tests in all assays combined (*n* = 5520)

Result	Active					Weak activity					Inactive					Sum	
Cultivation conditions	Low temp–low light	Low temp–high light	High temp–low light	High temp–high light	Total	Low temp–low light	Low temp–high light	High temp–low light	High temp–high light	Total	Low temp–low light	Low temp–high light	High temp–low light	High temp–high light	Total	Total	Hit rate %
Species: assay																	
*A. longicornis*	9		12	18	39	4		1	5	10	347		347	337	1031	1080	3.6 %
Melanoma	2		4	5	11	–	–	–	–	–	38	–	36	35	109	120	9.2 %
*E. coli*	–	–	–	–	–	–	–	–	–	–	40	–	40	40	120	120	0.0 %
*E. faecalis*	–	–	–	–	–	–	–	–	2	2	40	–	40	38	118	120	0.0 %
FRAP	–	–	–	–	–	–	–	–	–	–	40	–	40	40	120	120	0.0 %
MRSA	–	–	–	1	1	–	–	–	1	1	40	–	40	38	118	120	0.8 %
*P. aeruginosa*	–	–	–	–	–	–	–	–	–	–	40	–	40	40	120	120	0.0 %
Diabetes (PTP1B)	7	–	7	9	23	2	–	1	1	4	31	–	32	30	93	120	19.2 %
*S. aureus*	–	–	–	1	1	–	–	–	1	1	40	–	40	38	118	120	0.8 %
Immunomodulating	–	–	1	2	3	2	–	–	–	2	38	–	39	38	115	120	2.5 %
*C. furcellatus*	5	8	4	2	19	1	2	1	4	8	354	350	355	354	1413	1440	1.3 %
Melanoma	–	5	–	–	5	–	2	–	–	2	40	33	40	40	153	160	3.1 %
*E. coli*	–	–	–	–	–	–	–	–	–	–	40	40	40	40	160	160	0.0 %
*E. faecalis*	–	–	–	–	–	–	–	–	–	–	40	40	40	40	160	160	0.0 %
FRAP	–	–	–	–	–	–	–	–	–	–	40	40	40	40	160	160	0.0 %
MRSA	–	–	–	–	–	–	–	–	–	–	40	40	40	40	160	160	0.0 %
*P. aeruginosa*	–	–	–	–	–	–	–	–	–	–	40	40	40	40	160	160	0.0 %
Diabetes (PTP1B)	5	3	4	2	14	1	–	1	4	6	34	37	35	34	140	160	8.8 %
*S. aureus*	–	–	–	–	–	–	–	–	–	–	40	40	40	40	160	160	0.0 %
Immunomodulating	–	–	–	–	–	–	–	–	–	–	40	40	40	40	160	160	0.0 %
*C. socialis*	–	2	12	–	14	–	1	5	–	6	–	317	303	–	620	640	2.2 %
Melanoma	–	–	3	–	3	–	–	–	–	–	–	40	37	–	77	80	3.8 %
*E. coli*	–	–	–	–	–	–	–	–	–	–	–	40	40	–	80	80	0.0 %
*E. faecalis*	–	–	–	–	–	–	–	–	–	–	–	40	40	–	80	80	0.0 %
FRAP	–	2	–	–	2	–	–	–	–	–	–	38	40	–	78	80	2.5 %
MRSA	–	–	–	–	–	–	–	–	–	–	–	40	40	–	80	80	0.0 %
*P. aeruginosa*	–	–	–	–	–	–	–	–	–	–	–	40	40	–	80	80	0.0 %
Diabetes (PTP1B)	–	–	9	–	9	–	1	5	–	6	–	39	26	–	65	80	11.3 %
*S. aureus*	–	–	–	–	–	–	–	–	–	–	–	40	40	–	80	80	0.0 %
*P. glacialis*	14	5	7	–	26	5	7	3	–	15	341	348	350	360	1399	1440	1.8 %
Melanoma	3	–	3	–	6	–	–	–	–	–	37	40	37	40	154	160	3.8 %
*E. coli*	–	–	–	–	–	–	–	–	–	–	40	40	40	40	160	160	0.0 %
*E. faecalis*	–	–	–	–	–	–	–	–	–	–	40	40	40	40	160	160	0.0 %
FRAP	2	–	–	–	2	–	–	–	–	–	38	40	40	40	158	160	1.3 %
MRSA	–	–	–	–	–	–	–	–	–	–	40	40	40	40	160	160	0.0 %
*P. aeruginosa*	–	–	–	–	–	–	–	–	–	–	40	40	40	40	160	160	0.0 %
Diabetes (PTP1B)	1	1	4	–	6	2	–	3	–	5	37	39	33	40	149	160	3.8 %
*S. aureus*	–	–	–	–	–	–	–	–	–	–	40	40	40	40	160	160	0.0 %
Immunomodulating	8	4	–	–	12	3	7	–	–	10	29	29	40	40	138	160	7.5 %
*S. marinoi*	–	4	–	1	5	–	–	–	2	2	–	316	280	317	913	920	0.5 %
Melanoma	–	2	–	–	2	–	–	–	1	1	–	38	40	39	117	120	1.7 %
*E. coli*	–	–	–	–	–	–	–	–	–	–	–	40	40	40	120	120	0.0 %
*E. faecalis*	–	–	–	–	–	–	–	–	–	–	–	40	40	40	120	120	0.0 %
FRAP	–	2	–	1	3	–	–	–	–	–	–	38	–	39	77	80	3.8 %
MRSA	–	–	–	–	–	–	–	–	–	–	–	40	40	40	120	120	0.0 %
*P. aeruginosa*	–	–	–	–	–	–	–	–	–	–	–	40	40	40	120	120	0.0 %
Diabetes (PTP1B)	–	–	–	–	–	–	–	–	1	1	–	40	40	39	119	120	0.0 %
*S. aureus*	–	–	–	–	–	–	–	–	–	–	–	40	40	40	120	120	0.0 %
Sum	28	19	35	21	103	10	10	10	11	41	1042	1331	1635	1368	5376	5520	1.9 %

The results are reported as active, weakly active and inactive. There were a total of 103 hits

**Table 5:** Percentage of active fractions in the assays (*n* = 5520)

Assay	Active + weak active (%)	Active (%)
Melanoma	4.7	4.2
*E. coli*	0.0	0.0
*E. faecalis*	0.3	0.0
FRAP	1.2	1.2
MRSA	0.3	0.2
*P. aeruginosa*	0.0	0.0
Diabetes (PTP1B)	11.6	8.1
*S. aureus*	0.3	0.2
Immunomodulating	6.1	3.4
All assays	2.6	1.9

**Table 6:** Fisher’s exact test matrix showing *P* values of difference between each species and their counts of active vs. non-active HPLC fractions

Species	*A. longicornis*	*C. furcellatus*	*C. socialis*	*P. glacialis*	*S. marinoi*
*A. longicornis*	–	0.0002*	0.1127	0.0052*	0.0000*
*C. furcellatus*	0.0002*	–	0.1812	0.2971	0.0907
*C. socialis*	0.1127	0.1812	–	0.6044	0.0043*
*P. glacialis*	0.0052*	0.2971	0.6044	–	0.0086*
*S. marinoi*	0.0000*	0.0907	0.0043*	0.0086*	–

Significant results (*P* < 0.05) indicated with an asterisk (*)

**Fig. 2:**
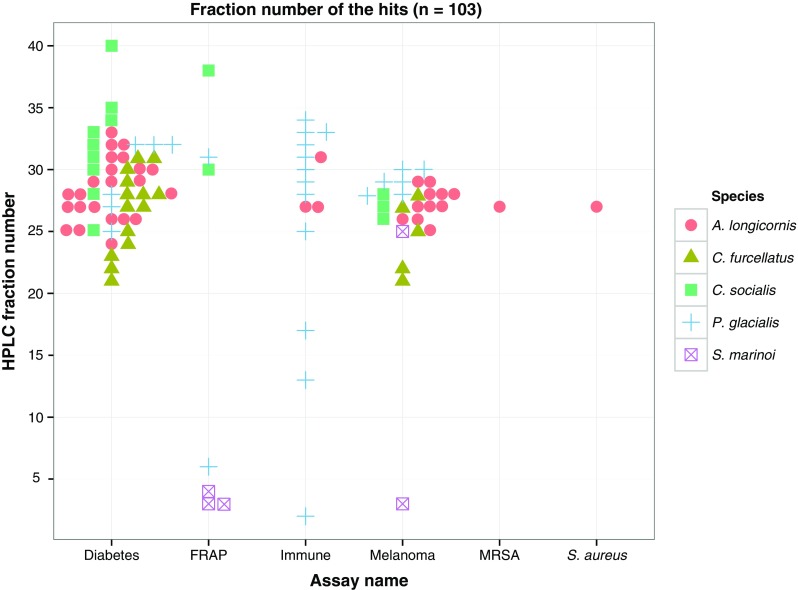
HPLC fraction number of hits (*n* = 103). The *x*-axis is the six assays with detected hits, and the *shapes* indicate species. Most of the hits were detected between fractions 25 and 35

In Table 7, the difference in hits vs. inactive between the different cultivation conditions is tested with pairwise Fisher’s exact tests and the only significant difference was between the low temperature–low light and low temperature–high light conditions. A chi-square test of independence between the total counts of active vs. inactive from the four cultivation conditions is not significant (*P* = 0.1). Looking closer at the count of hits vs. inactive at the two low temperature conditions vs. high temperature conditions reveals no significant differences (*P* = 0.76, Fisher’s exact test). However, there was a significant difference between the two low light and the two high light conditions regarding counts of hits vs. inactive (*P* = 0.028, Fisher’s exact test), with low light being the condition producing the highest number of hits (*n* = 63). Figure 2 shows an overview of fraction number of all hits in relation to species and assay. Most of the active fractions were between fraction number 25 and 35 in the anti-cancer and the diabetes assay.

**Table 7:** Fisher’s exact test matrix showing *P* values of difference between the count of active vs. non-active in each cultivation condition

Cultivation condition	Low temp–low light	Low temp–high light	High temp–low light	High temp–high light
Low temp–low light	–	0.0375*	0.4333	0.0586
Low temp–high light	0.0375*	–	0.169	0.8742
High temp–low light	0.4333	0.169	–	0.2786
High temp–high light	0.0586	0.8742	0.2786	–

Significant results (*P* < 0.05) indicated with an asterisk (*)

### *Attheya longicornis*

Extracts of *A. longicornis* from all three cultivation conditions contained fractions active against cancer cells, and this was the only species that was active against cancer cells at cultivation conditions it was cultivated at. Extracts from *A. longicornis* did also have activity against MRSA and *S. aureus* and had a weak activity against *E. faecalis*, but it was only cultures exposed to high light and high temperature that had this activity. Thus, *A. longicornis* was the only diatom in this study that was active against bacteria. In the anti-inflammatory assay, extracts from all cultivation conditions except the low temp–low light condition were active, with *A. longicornis* cultivated at high temp–high light showing the strongest activity. All extracts of *A. longicornis* were active against diabetes II. There was no FRAP anti-oxidant activity in the extracts of *A. longicornis*.

### *Chaetoceros furcellatus*

The only *C. furcellatus* extract with activity against cancer cells was cultivated at low temperature and high light. We found no activity against bacteria, inflammation or anti-oxidants (as measured with FRAP) in the *C. furcellatus* extracts. Anti-diabetes II activity was detected in all *C. furcellatus* extracts.

### *Chaetoceros socialis*

We only had data from two cultivation conditions with *C. socialis*. However, extract from the high temperature–low light were active against cancer cells, while no fractions in the extract from low temperature–high light condition had anti-cancer activity. *C. socialis* showed no anti-inflammatory activity. Two fractions from the extract of *C. socialis* cultivated at low temperature–high light were active in the FRAP assay. The extract of *C. socialis* cultivated at high temperature–low light was active against diabetes II.

### *Porosira glacialis*

Both low light cultivations (high and low temperature) gave rise to extracts with anti-cancer activity. The *P. glacialis* extracts were not active against bacteria. The two extracts from low temperature contained fractions active in the anti-inflammatory assay. In the extract of *P. glacialis* cultivated at low temperature–low light, we detected two fractions (6 and 31) with notable activity in the FRAP assay. We also found anti-diabetes II activity in *P. glacialis* extracts from all but the “high temp–high light” cultivation condition. The *P. glacialis* cultivated at high temp–high light produced no bioactive fractions.

### *Skeletonema marinoi*

The *S. marinoi* low temperature–low light cultivation failed. The extract from *S. marinoi* cultivated at low temperature–high light was active against cancer cells, and there was anti-oxidant activity in all the three *S. marinoi* extracts. We found no activity against bacteria, inflammation or diabetes II in the extracts of *S. marinoi*. Notably, it was the only species from which we did not detect anti-diabetes II activity.

## Discussion

In the present study, we investigated five northern diatoms for bioactivity and whether we could get variation in bioactivity by changing light and temperature. All diatoms except *S. marinoi* showed activity in the diabetes assay. Though the activity in the diabetes assay could be caused by the pigment fucoxanthin that is present in diatoms and has been shown to be a potent inhibitor of PTP1B (Jung et al. 2012), which was the same enzyme we measured inhibition of in the diabetes assay we used. The five diatoms screened were active in the anti-cancer assay (melanoma), but not at all cultivation conditions except for *A. longicornis*. Furthermore, there was species-specific difference in relation to cultivation condition and anti-cancer activity. The overall bioactivity was significantly different between species, with *A. longicornis* being the species producing the highest number of hits (*n* = 39) and *S. marinoi* the lowest (*n* = 5). However, keep in mind that the cultivation conditions we applied were different between these species (see Table 1), so they are not directly comparable. For every therapeutic area, we screened our extracts in a minimum of one and a maximum of four of the five species yielded *different* results between cultivation conditions. Regarding the effect of temperature upon number of active fractions vs. inactive, we found only a minor difference in hits between high and low cultivation temperature and it was not statistically significant, though the variation was in both directions, thus drawing the statistical significance level down. In other words, there was no given temperature producing more hits. Nevertheless, metabolite profiling of diatoms has previously shown that there are large numbers of low-molecular weight compounds present in diatom biomass, and it is known that the diatom metabolome can vary with temperature (Huseby et al. 2013; Nappo et al. 2009). Light, on the other hand, had a significant effect on the total number hits, with the low light conditions resulting in a higher number of hits than the high light condition. It was especially interesting that anti-bacterial activity against the Gram-positive bacteria MRSA, *S. aureus* and *E. faecalis*, was only detected in the extract subjected to high light. In its natural habitat, *A. longicornis* is an epiphytic, facultative psychrophilic alga able to thrive even in the polar drift ice (Orlova et al. 2002). And at the lower irradiances used in this experiment, *A. longicornis* grew well with no visible signs of stress. Hence, the lack of anti-bacterial activity at the lower irradiances did not seem to be caused by a general physiological decline, though we cannot rule out that some metabolic pathways may be downregulated in order to economize with the limited available energy. Another example of variability, *P. glacialis*, was most active at low temperature and low light with anti-cancer, anti-inflammatory, anti-diabetes and FRAP activities detected, while at high temperature, it was only active against cancer cells and diabetes. The fact that no activity was found in the extract from the biomass cultivated at high temperature and high light indicates substantial changes in its chemical composition when subjected to different environmental conditions. We did not attempt to identify any compounds responsible for bioactivity and it is likely that some compounds were responsible for activity in more than one species and/or in more than one assay. It is also possible that some cultivation conditions caused an increased production of some compounds that otherwise would be present in amounts below the activity threshold. Even the two closely related species, *C. socialis* and *C. furcellatus*, did not produce identical results regarding the bioactivities detected in their extracts (Fig. 1) even though the overall difference in total bioactivity was insignificant (Table 6). Due to inherent challenges related to large-scale cultivation, we cannot rule out that some of the variations observed were influenced by other factors beyond our control, such as life cycle stages, unknown random effects, density dependent factors, seasonal seawater variations or signal molecules therein. However, we were aware of and strived to minimize these effects. Light and temperature are important environmental cues in the natural habitats of phytoplankton and are affecting many biochemical pathways and reactions and thus also bioactivity. All in all, our experiment attested that diatom clonal culture bioactivity could be variable.

Observed and reported chemical compositions and production of both biomass and secondary metabolites of microalgae are not static. Instead, the production of primary and secondary metabolites is clearly variable, regulated or modulated by, e.g. light (Depauw et al. 2012), temperature (Thomson et al. 1992; Huseby et al. 2013), growth phase stage (Barofsky et al. 2009, 2010; Vidoudez and Pohnert 2012), strain or species (Degerlund et al. 2012), grazing (Pohnert 2002), culture media (Alkhamis and Qin 2015), extraction method (Jüttner 2001), freezing and thawing of samples (Eilertsen et al. 2014), clonal variability (Gerecht et al. 2011) various stress and probably many other factors (Chen et al. 2011). This variability complicates interpretation of results and makes it very difficult to make general statements about which factor controls production of, e.g. bioactive metabolites, as the numerous possible random effects or ghost effects may easily distort results. On the other hand, metabolic plasticity is *exactly* what is needed if one wants to increase the probability of discovering novel bioactive metabolites without having to build an extensive culture collection. Because if you have organisms with metabolic plasticity, you can simply cultivate the organisms at several different conditions and hopefully your organism produce interesting chemistry at some of these cultivation conditions. This strategy, termed OSMAC (One strain–many compounds), has successfully been applied for biodiscovery in bacteria (Bode et al. 2002) and could potentially also work on diatoms.

In conclusion, the bioactivity in our experiment was variable and possibly modified by light and temperature regimes used in the cultivation, though our data does not reveal any clear trends that could be applied as a standard for cultivation of other diatom species with the aim of getting bioactive biomass. One simply needs to perform batch cultivation with e.g. varying light and temperature regimes in order to trigger or increase production of desired metabolites. This study indicates that more bioactive compounds could potentially be gained from single isolates of diatoms by repeatedly cultivating the isolates with more than just one fixed set of cultivation conditions, and similar approaches has been applied in e.g. the studies by Zupo et al. (2011) and Mouget et al. (2005). Lastly, light and temperature are only two factors and many other factors could be adjusted to trigger cryptic bioactivity in diatoms and hopefully lead to discovery of novel bioactive compounds.

## References

[CR1] Alkhamis Y, Qin JG (2015). Comparison of pigment and proximate compositions of *Tisochrysis lutea* in phototrophic and mixotrophic cultures. J Appl Phycol.

[CR2] Armbrust E, Berges JA, Bowler C, Green BR, Martinez D, Putnam NH, Zhou S, Allen AE, Apt KE, Bechner M, Brzezinski MA, Chaal BK, Chiovitti A, Davis AK, Demarest MS, Detter JC, Glavina T, Goodstein D, Hadi MZ, Hellsten U, Hildebrand M, Jenkins BD, Jurka J, Kapitonov VV, Kröger N, Lau WWY, Lane TW, Larimer FW, Lippmeier JC, Lucas S, Mónica M, Montsant A, Obornik M, Parker MS, Pelenik B, Pazour GJ, Richardson PM, Rynearson TA, Saito MA, Schwartz DC, Thamatrakoln K, Valentin K, Vardi A, Wilkerson FP, Rokhsar DS (2004). The genome of the diatom *Thalassiosira pseudonana*: ecology, evolution, and metabolism. Science.

[CR3] Barofsky A, Vidoudez C, Pohnert G (2009). Metabolic profiling reveals growth stage variability in diatom exudates. Limnol Oceanogr Methods.

[CR4] Barofsky A, Simonelli P, Vidoudez C, Troedsson C, Nejstgaard JC, Jakobsen HH, Pohnert G (2010). Growth phase of the diatom *Skeletonema marinoi* influences the metabolic profile of the cells and the selective feeding of the copepod *Calanus* spp. J Plankton Res.

[CR5] Benzie IFF, Strain JJ (1996). The ferric reducing ability of plasma (FRAP) as a measure of “antioxidant power”: the FRAP assay. Anal Biochem.

[CR6] Bergé J-P, Bourgougnon N, Alban S, Pojer F, Billaudel S, Chermann J-C, Robert J, Franz G (1999). Antiviral and anticoagulant activities of a water-soluble fraction of the marine diatom *Haslea ostrearia*. Planta Med.

[CR7] Blunt JW, Copp BR, Hu W-P, Munro MH, Northcote PT, Prinsep MR (2009). Marine natural products. Nat Prod Rep.

[CR8] Blunt JW, Copp BR, Munro MH, Northcote PT, Prinsep MR (2010). Marine natural products. Nat Prod Rep.

[CR9] Blunt JW, Copp BR, Munro MH, Northcote PT, Prinsep MR (2011). Marine natural products. Nat Prod Rep.

[CR10] Bode HB, Bethe B, Höfs R, Zeeck A (2002). Big effects from small changes: possible ways to explore nature’s chemical diversity. ChemBioChem.

[CR11] Bull AT, Stach JEM (2007). Marine actinobacteria: new opportunities for natural product search and discovery. Trends Microbiol.

[CR12] Buttino I, De Rosa G, Carotenuto Y, Mazzella M, Ianora A, Esposito F, Vitiello V, Quaglia F, La Rotonda MI, Miralto A (2008). Aldehyde-encapsulating liposomes impair marine grazer survivorship. J Exp Biol.

[CR13] Carbonnelle D, Pondaven P, Morançais M, Massé G, Bosch S, Jacquot C, Briand G, Robert J, Roussakis C (1999). Antitumor and antiproliferative effects of an aqueous extract from the marine diatom *Haslea ostrearia* (Simonsen) against solid tumors: lung carcinoma (NSCLC-N6), kidney carcinoma (E39) and melanoma (M96) cell lines. Anticancer Res.

[CR14] Chen C-Y, Yeh K-L, Aisyah R, Lee D-J, Chang J-S (2011). Cultivation, photobioreactor design and harvesting of microalgae for biodiesel production: a critical review. Bioresour Technol.

[CR15] Degerlund M, Huseby S, Zingone A, Sarno D, Landfald B (2012). Functional diversity in cryptic species of *Chaetoceros socialis* Lauder (Bacillariophyceae). Plankton Res.

[CR16] Depauw FA, Rogato A, d’Alcalá MR, Falciatore A (2012). Exploring the molecular basis of responses to light in marine diatoms. J Exp Bot.

[CR17] Eilertsen HC, Huseby S, Degerlund M, Eriksen GK, Ingebrigtsen RA, Hansen E (2014). The effect of freeze/thaw cycles on reproducibility of metabolic profiling of marine microalgal extracts using direct infusion high-resolution mass spectrometry (HR-MS). Molecules.

[CR18] Field CB, Behrenfeld MJ, Randerson JT, Falkowski P (1998). Primary production of the biosphere: integrating terrestrial and oceanic components. Science.

[CR19] Folmer F, Jaspars M, Dicato M, Diederich M (2010). Photosynthetic marine organisms as a source of anticancer compounds. Phytochem Rev.

[CR20] Gerecht A, Romano G, Ianora A, d’Ippolito G, Cutignano A, Fontana A (2011). Plasticity of oxylipin metabolism among clones of the marine diatom *Skeletonema marinoi* (Bacillariophyceae). J Phycol.

[CR21] Guedes Catarina A, Amaro HM, Sousa-Pinto I, Xavier Malcata F, Hernándes-Ledesma B, Herrero M (2013). Bioactive carotenoids from microalgae. Bioactive compounds from marine foods: plant and animal sources.

[CR22] Haimeur A, Ulmann L, Mimouni V, Guéno F, Pineau-Vincent F, Meskini N, Tremblin G (2012). The role of *Odontella aurita*, a marine diatom rich in EPA, as a dietary supplement in dyslipidemia, platelet function and oxidative stress in high-fat fed rats. Lipids Health Dis.

[CR23] Hamm CE, Merkel R, Springer O, Jurkojc P, Maier C, Prechtel K, Smetacek V (2003). Architecture and material properties of diatom shells provide effective mechanical protection. Nature.

[CR24] Harrison PJ, Conway HL, Holmes RW, Davis CO (1977). Marine diatoms grown in chemostats under silicate or ammonium limitation. III. Cellular chemical composition and morphology of *Chaetoceros debilis*, *Skeletonema costatum*, and *Thalassiosira gravida*. Mar Biol.

[CR25] Huseby S, Degerlund M, Eriksen GK, Ingebrigtsen RA, Eilertsen HC, Hansen E (2013) Chemical diversity as a function of temperature in six northern diatom species. Mar Drugs 11:4232–4245 10.3390/md11114232 PMC3853725 24177671

[CR26] Ianora A, Poulet SA, Miralto A (2003) The effects of diatoms on copepod reproduction: a review. Phycologia 42:351–363

[CR27] Jung HA, Islam MN, Lee CM, Jeong HO, Chung HY, Woo HC, Choi JS (2012). Promising antidiabetic potential of fucoxanthin isolated from the edible brown algae *Eisenia bicyclis* and *Undaria pinnatifida*. Fish Sci.

[CR28] Jüttner F (2001). Liberation of 5, 8, 11, 14, 17‐eicosapentaenoic acid and other polyunsaturated fatty acids from lipids as a grazer defense reaction in epilithic diatom biofilms. J Phycol.

[CR29] Lakeman MB, von Dassow P, Cattolico RA (2009). The strain concept in phytoplankton ecology. Harmful Algae.

[CR30] Lebeau T, Robert J-M (2003). Diatom cultivation and biotechnologically relevant products. Part II: current and putative products. Appl Microbiol Biotechnol.

[CR31] Lind KF, Hansen E, Østerud B, Eilertsen K-E, Bayer A, Engqvist M, Leszczak K, Jørgensen TØ, Andersen JH (2013). Antioxidant and anti-inflammatory activities of barettin. Mar Drugs.

[CR32] Moreau D, Tomasoni C, Jacquot C, Kaas R, Le Guedes R, Cadoret J-P, Muller-Feuga A, Kontiza I, Vagias C, Roussis V (2006). Cultivated microalgae and the carotenoid fucoxanthin from *Odontella aurita* as potent anti-proliferative agents in bronchopulmonary and epithelial cell lines. Environ Toxicol Pharmacol.

[CR33] Mouget J-L, Rosa P, Vachoux C, Tremblin G (2005). Enhancement of marennine production by blue light in the diatom *Haslea ostrearia*. J Appl Phycol.

[CR34] Nappo M, Berkov S, Codina C, Avila C, Messina P, Zupo V, Bastida J (2009). Metabolite profiling of the benthic diatom *Cocconeis scutellum* by GC-MS. J Appl Phycol.

[CR35] Nelson DM, Treguer P, Brzezinski MA, Leynaert A, Queguiner B (1995). Production and dissolution of biogenic silica in the ocean: revised global estimates, comparison with regional data and relationship to biogenic sedimentation. Global Biogeochem Cycles.

[CR36] Orlova TY, Stonik IV, Aizdaicher NA (2002). Morphology and biology of the diatom alga *Atteya longicornis* from the Sea of Japan. Russ J Mar Biol.

[CR37] Pohnert G (2002). Phospholipase A2 activity triggers the wound-activated chemical defense in the diatom *Thalassiosira rotula*. Plant Physiol.

[CR38] Prestegard SK, Oftedal L, Coyne RT, Nygaard G, Skjærven KH, Knutsen G, Døskeland SO, Herfindal L (2009). Marine benthic diatoms contain compounds able to induce leukemia cell death and modulate blood platelet activity. Mar Drugs.

[CR39] Rowland S, Belt S, Wraige E, Massé G, Roussakis C, Robert J-M (2001). Effects of temperature on polyunsaturation in cytostatic lipids of *Haslea ostrearia*. Phytochemistry.

[CR40] Smetacek V (2001). A watery arms race. Nature.

[CR41] Sultana B, Anwar F, Ashraf M (2009). Effect of extraction solvent/technique on the antioxidant activity of selected medicinal plant extracts. Molecules.

[CR42] Thomson PA, Guo MX, Harrison PJ, Whyte JNC (1992). Effects of variation in temperature. II. On the fatty acid composition of eight species of marine phytoplankton. J Phycol.

[CR43] Tredici MR, Richmond A (2004). Mass production of microalgae: photobioreactors. Handbook of microalgal culture. Biotechnology and applied phycology.

[CR44] van den Hoek C, Mann DG, Jahns HM (1995). Algae an introduction to phycology.

[CR45] Vidoudez C, Pohnert G (2012). Comparative metabolomics of the diatom *Skeletonema marinoi* in different growth phases. Metabolomics.

[CR46] Wickham H (2009) ggplot2: elegant graphics for data analysis. Springer Science & Business Media

[CR47] Zupo V, Patalano C, Messina P (2011). Culture conditions influence the growth dynamics and the production of *Cocconeis scutellum* (Bacillariophyta). J Phycol.

[CR48] Zupo V, Jüttner F, Maibam C, Butera E, Blom JF (2014). Apoptogenic metabolites in fractions of the benthic diatom *Cocconeis scutellum parva*. Mar Drugs.

